# How to Interact with a Fully Autonomous Vehicle: Naturalistic Ways for Drivers to Intervene in the Vehicle System While Performing Non-Driving Related Tasks

**DOI:** 10.3390/s21062206

**Published:** 2021-03-21

**Authors:** Aya Ataya, Won Kim, Ahmed Elsharkawy, SeungJun Kim

**Affiliations:** Gwangju Institute of Science and Technology, School of Integrated Technology, Gwangju 61005, Korea; aya.ataya@gm.gist.ac.kr (A.A.); kimwon30@gm.gist.ac.kr (W.K.); elsharkawy@gm.gist.ac.kr (A.E.)

**Keywords:** fully autonomous vehicle (FAV), input interactions, non-driving related task (NDRT), intervene vehicle system

## Abstract

Autonomous vehicle technology increasingly allows drivers to turn their primary attention to secondary tasks (e.g., eating or working). This dramatic behavior change thus requires new input modalities to support driver–vehicle interaction, which must match the driver’s in-vehicle activities and the interaction situation. Prior studies that addressed this question did not consider how acceptance for inputs was affected by the physical and cognitive levels experienced by drivers engaged in Non-driving Related Tasks (NDRTs) or how their acceptance varies according to the interaction situation. This study investigates naturalistic interactions with a fully autonomous vehicle system in different intervention scenarios while drivers perform NDRTs. We presented an online methodology to 360 participants showing four NDRTs with different physical and cognitive engagement levels, and tested the six most common intervention scenarios (24 cases). Participants evaluated our proposed seven natural input interactions for each case: touch, voice, hand gesture, and their combinations. Results show that NDRTs influence the driver’s input interaction more than intervention scenario categories. In contrast, variation of physical load has more influence on input selection than variation of cognitive load. We also present a decision-making model of driver preferences to determine the most natural inputs and help User Experience designers better meet drivers’ needs.

## 1. Introduction

Increased vehicle autonomy, driven largely by the development of Artificial Intelligence (AI), means that vehicle operation is no longer the driver’s primary task [1,2]. As vehicle automation levels rise from the Society of Automotive Engineers (SAE) Level 0 (no automation/the driver is fully responsible for driving) to Level 5 (fully autonomous vehicle), drivers are able to relinquish more vehicle control and engage in Non-Driving Related Tasks (NDRTs), such as relaxing, working on a laptop, or even sleeping [3,4,5,6,7]. This difference in activities may be accompanied by a change in position inside the Fully Autonomous Vehicle (FAV), for example, reclining the seat to use a laptop would position the driver farther from the dashboard than during normal driving [8,9]. Yet drivers engaged in NDRTs still need to interact with the vehicle [10] (e.g., adjust the infotainment system) or intervene to assume control (e.g., instruct the vehicle to pass a slower car ahead) [11,12,13]. Further, different NDRTs demand of drivers different physical and cognitive loads [9,14,15], which are also influenced by differences between maneuver- and nonmaneuver-based intervention scenarios [16,17]. Consequently, conventional touch and voice interactions may not be sufficient to accommodate these changes in physical and cognitive load thus do not adequately support drivers to perform different intervention scenarios.

While developments in interior design [5,9,18] were studied to better accommodate drivers, their interaction needs inside FAVs have yet to be fully addressed [10,19,20]. Researchers should consider the conditions of individual drivers and what they might need to do. In addition to the common touch interaction, remote interaction techniques such as voice, hand gesture, and gaze are particularly strong candidates to suit the FAV interaction environment. Thus, it is important to test these input interactions and suggest hybrid forms (e.g., voice + hand gesture, voice + touch, etc.) to suit all possible situations.

In nonautonomous vehicles, identifying the best input interactions often aims to increase safety by reducing driver distraction or cognitive demands [21,22,23]. Since FAVs integrate more technology and require a broader variety of interaction than nonautonomous vehicles [24], identifying the best input interactions will also enhance AV acceptance and allow drivers to seamlessly continue performing NDRTs safely, efficiently, and intuitively. In other words, these input interactions should be easy to learn and intuitive to use, and must not increase driver stress [18,19,20]. Our goal is to understand the most natural input interactions that allow drivers to perform multiple intervention scenarios while engaged in different levels of NDRTs. This study also investigates factors that influence driver selection of input interaction technique while considering their level of engagement in different NDRTs and various possible intervention scenarios to increase driver acceptance and satisfaction about user interface in autonomous vehicles. Based on these goals, we ask the following Research Questions (RQ):

- RQ1: What are the most and least preferable natural input interactions in FAVs regardless of NDRTs and intervention scenarios? How could FAVs help regulate interactions based on the driver’s in-vehicle activity and the driving context?
- RQ2: Based on previous studies [14,25,26,27] that show that physical and cognitive load negatively affect user interaction and influence human–computer interaction and design, how do NDRTs with various physical and cognitive engagement levels affect driver acceptance of input interactions inside FAVs?
- RQ3: As maneuver-based intervention is an essential technique since it gives AV drivers a feeling of control and satisfaction compared to pure automation [24,28], how do maneuver and nonmaneuver-based intervention scenarios affect a driver’s selection of input interactions? Additionally, which factor most influences the selection of input interactions inside FAVs?

To address these questions, we employed an online video-based comprehensive survey to efficiently present and test a large number of factors across a large number of participants [29,30,31]. The videos depict a driver involved in four NDRTs who is asked to perform six intervention scenarios using the suggested seven input interactions. Then, participants relate their impression and suggestions about the tested input interactions. These videos reflected a real-world driving environment to help participants immersive themselves in the scenario and better imagine themselves in the driver’s seat. Participants’ answers were then analyzed to formulate the following contributions:

1. Demonstrate the most naturalistic input interaction possibilities in FAVs for NDRTs, according to variation in physical and cognitive load demands.
2. Understand the factors that affect a driver’s natural input interaction while intervening with the FAV system and highlight their impacts.
3. Suggest guidelines to design natural input interaction channels for FAVs based on the driver’s level of engagement in NDRTs and the specific intervention scenario. In other words, design a mental model to represent the driver’s method of selecting the most appropriate input interaction inside the FAV.

### 1.1. Literature Review

In nonautonomous vehicles, the driver controls the vehicle throughout the trip and interactions with the vehicle tend toward nonmaneuver-based interventions, such as using infotainment or a navigation system [21]. With increasing levels of vehicle autonomy, maneuver control, such as steering, braking, or stopping, is still needed, though not at all times, and AVs can still hand over control to the driver [32,33,34]. In a Level 5 (SAE) vehicle, neither traditional maneuver control nor takeover ability exists [13,16]. However, having the ability to control the vehicle was identified as a leading factor in AV acceptance [15,20,28,35,36]. Roedel et al. [37] found that inexperienced, young drivers show low acceptance for AVs as they feel bored and out of control.

Feelings of control in the AV rely on effective communication with the vehicle. For lower-level automation (SAE Levels 2 and 3) [1], maneuver-based driving was introduced to keep the driver in the loop by controlling or cooperating with the vehicle to guide and handle system limitations rather than hand over control [38,39]. Similarly, in higher-level automation, maneuver-based intervention has been proposed as the way for drivers to communicate with the FAV in order to intervene in the automated system to execute the desired maneuver, such as suddenly stopping the vehicle [13,16,40]. Maneuver-based intervention improves trust and acceptance of the AV by keeping the driver in control [13,41], yet applying these interventions in FAVs requires new input control channels to execute the command, as steering wheels and pedals are not designed to be used for this purpose. Previous studies [16,17,42] have discussed intervention scenarios where the driver wants to spontaneously influence FAV behavior (e.g., stop the car to pick up a friend, overtake the car ahead, or select a parking lot). These studies have focused on intervention scenarios based solely on controlling vehicle driving (maneuver-based driving), which, unfortunately, may not cover all possible interaction aspects [43]. Previous studies have shown that drivers are most interested in performing the following NDRTs (listed in order of preference): using multimedia, such as a phone or tablet; working on a laptop; relaxing; eating; and sleeping [3,4,5,6,7,44]. Similarly, manual driving studies have investigated how the physical and cognitive demands of NDRTs affect driving quality [15,26,45,46]. For instance, eating and holding a phone have been shown to differently affect a driver’s physical load and require different levels of cognitive and physical engagement [14,15,45].

In FAVs, maintaining vehicle control is no longer the driver’s main task. As there is no possibility for a takeover request from the FAV, drivers can freely and confidently engage in NDRTs [6]. This study [47] has tested only one NDRT (using a smart phone) on overall participant preference order for the tested input interactions (voice, touch, gaze-head) while sampling driver experience about the AV action.

Even though performing NDRTs was found as a major advantage of FAVs [36,48], previous studies have not considered how different NDRTs affect drivers’ acceptance of input interaction [16,17]. In manual driving, where driving itself is the primary task, typical input interactions (e.g., buttons, touch, voice, and hand gestures) are designed to increase safety rather than improve performance [21,23,45,49]. Previous AV studies have investigated the use of touch panels for drivers to select one of the vehicle’s suggested propositions (e.g., pass the car ahead or hand over driving control) when the automated system has reached its limit [32,34], or to select AV maneuvers (e.g., change lanes) via touch input implemented on the steering wheel [33]. However, these approaches require the driver’s hands to be free to interact with the AV—a less-than-ideal condition when engaged in an NDRT—and that the driver be close enough to the steering wheel to reach the touch panel. Further, some participants did not prefer touch input because they had to switch their eyes from the road view to the touch interface while interacting [32].

In addition to touch interaction, hand gesture has been applied for maneuver-based intervention [16] and to control in-car lateral and longitudinal AV motions [12], showing promising usability as a remote input interaction in both studies. Still, problems persist with overlapping commands and rates of misrecognition, and drivers struggle to remember each hand gesture. Automotive researchers have tried to solve these problems by proposing a multimodal input that combines eye gaze and hand gesture inputs, where gaze is used to localize the target and hand gesture to coordinate pointing [21,23]. The combined multimodal input has shown promise as users can interact with a wide range of vehicle functions without having to learn new gestures.

Voice input can supplement the drawbacks of touch or hand gesture inputs by supporting remote and hands-free input interactions. Voice input, one of the most common input interactions in today’s vehicles and AV studies, has been implemented to support driver–vehicle cooperation and select vehicle maneuvers. Drivers prefer voice to touch input [32,47] and hand gestures [16], since voice commands do not require physical movement and allow them to keep their eyes on the road during hands-free interaction. However, voice input may not work well in a noisy environment (e.g., group conversations), and drivers may not completely trust speech recognition or may become confused about the appropriate command to initiate a desired action [10,16].

To simplify voice input commands, AV and non-AV studies suggest voice multimodal inputs such as voice + touch or voice + hand gesture [17,21,22,50,51]. While participants reported that voice + hand gesture was more natural, intuitive, and less cognitively demanding than voice + touch [17], the latter was preferred for its shorter interaction generation [21]. In addition, voice + gaze has been shown to reduce interaction duration as compared to voice as a single input modality [21,22].

Despite these findings, Natural User Interface (NUI) studies have not considered the effects of the cognitive and physical load of NDRTs on the user’s preference of interface in FAVs [16,17]. Moreover, since each input interaction has its own cognitive and physical load (e.g., voice input increases cognitive load, touch input increases physical load), the various conditions of NDRTs may impact the driver’s interaction behavior with FAVs [20].

### 1.2. Proposed Input Interactions, NDRTs, and Intervention Scenarios

**Figure 1:**
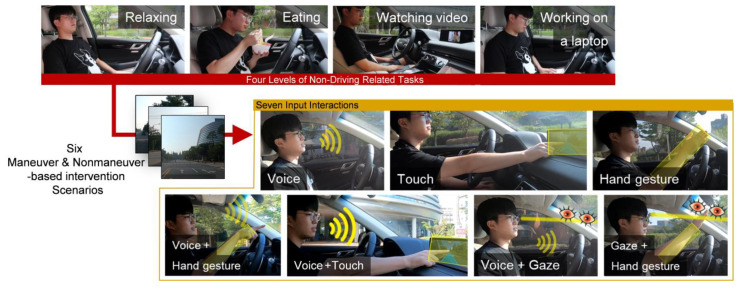
Which input interaction is most naturalistic? We analyzed preferences of seven input interactions associated with the proposed non-driving related tasks and intervention scenarios.

**Proposed Input Interactions:** This present study explores both single and multimodal NUI input interactions to determine how different NDRTs affect driver acceptance for natural input interaction [16,17,21,52]. Those inputs include voice, touch, hand gesture, voice + touch (e.g., where touch is used to select the object and voice input to trigger the selection), voice + gaze, voice + hand gesture, and gaze + hand gesture, see Figure 1.

**Proposed Non-driving Related Tasks:** We investigated the most frequent NDRTs in an AV to assess how their physical and cognitive loads affect a driver’s input interactions [4,5,6]. The NDRTs were categorized in descending order (high to low) based on physical and cognitive load [9,26,45]. For example, previous studies have shown that *watching a video* NDRT creates a significant negative correlation between mental demand and takeover time [15]. Therefore, our study considers *watching a video* as an example of a high cognitive load NDRT (Table 1).

**Proposed Intervention Scenarios:** Our study presented six intervention scenarios that cover both Maneuver-Based Interventions (MBIs) (four scenarios) and Nonmaneuver-Based Interventions (NMBI) (two scenarios). MBIs include lateral control, longitudinal control, stopping the vehicle at a designated area [12,17,53], and rerouting the navigation [18]. NMBIs include requesting information (e.g., about an interesting building or available parking near the final destination) [12,17,53] which is then shown on the head-up display, the most preferred display [25,54], and controlling in-vehicle features, such as playing a video using the in-vehicle infotainment system [11,21], (Table 2). Our proposed scenarios were parameterized in the perspective of driver intervention in the FAV to control vehicle movement (MBI) or for entertainment (NMBI).

The paper is organized as follows: Section 2 discusses key steps for structuring and conducting our video-based survey, subjects’ demographic characteristics, and explains how we filtered our data. Section 3 reviews our survey data results. Section 4 discusses our findings, proposes a decision-making model, and acknowledges the limitations of our study. Lastly, Section 5 presents our conclusion and discusses future work.

## 2. Method

Many AV studies rely on survey-based video, which allows researchers to test more elements with a larger number of responses than other qualitative methods [30,55]. The methodology can breach the communication barrier between participant and researcher and, compared to text-only surveys, allows researchers to collect more quantitatively and qualitatively complete data. It also prompts participants to be more relaxed and aware and thus heightens their sense of immersion [56]. For these reasons, we used a survey-based video to investigate a wide range of factors that influence driver preferences of input interaction techniques.

Stanton et al. [57] presented lane change examples in 60 videos to determine whether participants could distinguish between AV behavior and manual driving behavior. In a similar study, [30] presented scenarios about AV behaviors to collect user-centered perceptions about how they would like semi-AVs to act and alert them of the vehicles’ decisions. For vehicle-to-pedestrian displays, [31] presented a 30 creative design concepts to a large number of participants. Moreover, Fuest et al. [58] compared a video-based method with a real world experiment regarding communication between AVs and pedestrians and found that both were in agreement. Following these findings, we employed comprehensive survey-based videos that present NDRTs with different levels of physical and cognitive load and various intervention scenarios to understand participants’ preferences and acceptance about the suggested input interactions, and how their preference is influenced by the variances in NDRTs and intervention scenarios.

Our video-based survey was distributed using Amazon Mechanical Turk (MTurk), an online recruiting and crowdsourcing platform in which workers (known as MTurkers) accept and complete a Human Intelligence Task (HIT), or a single, self-contained task. For each completed HIT, the MTurker collects a reward. Compared to traditional recruitment methods, the MTurk platform has been found to be efficient for recruiting diverse, more representative participants and to deliver higher speed of completion at a lower price [59,60]. MTurk has supported many researchers in collecting a wide range of responses while providing options to control a specific characteristic (e.g., age or education level) in the recruited participants from suggested categories [56,60].

### 2.1. Procedure

To achieve our survey goal, in a pilot test, we recruited 20 participants to complete our survey and interviewed them about whether our videos and questionnaires delivered our concept clearly. In order to avoid participant fatigue and maintain their engagement, we designed short videos so that the total survey time did not exceed 1 h [61]. The pilot test confirmed that the total survey time was between 25 and 30 min. Based on participant feedback, we revised unclear points in the survey and set a completion time of 1 h for each survey.

Next, we recruited 360 participants using MTurk. Participant ages ranged from 18 to 70 years (*M* = 37.08, *SD* = 10.2; Gender: Male = 268, Female = 92). Drivers’ population is too large; therefore, selecting a large research sample that achieves a high confidence level with a low margin of error is challenging. In relation, an online survey was designed to study AV-to-pedestrian interactions [31], where 200 participants were recruited. Another study recruited 300 participants to check how drivers prefer the semiautonomous vehicle to inform them about the vehicle’s decisions in different situations [30]. Therefore, we infer that a sample between 200 to 300 participants or more is sufficient for further statistical research, where acceptable confidence level and margin of error can be achieved.

Using a between group design, 120 answers were received for each intervention scenario. Seventy-two percent of participants had knowledge of or experience with Advanced Driver Assistance Systems (ADAS; e.g., forward collision alert, lane departure alert, automatic emergency braking, pedestrian collision alert) while 28% did not. Each participant was compensated approximately 7 USD after completing the survey.

To guarantee data quality and reliable participants [59,62], we screened our data by specifying additional qualifications for our participants. Participants must be master MTurkers who have achieved a HIT approval rate greater than 95% positive with a minimum of 1000 previous HITs. To ensure that they were experienced drivers, we also specified that each participant had to own a car, be aware of our tested driving scenarios, and be familiar with input interactions in today’s in-vehicle infotainment systems (e.g., touch, voice). We informed participants their responses would be used for a research study and each answer would be carefully checked, which [59] found reduces invalid answers and increases time on task.

### 2.2. Survey Design

**Figure 2:**
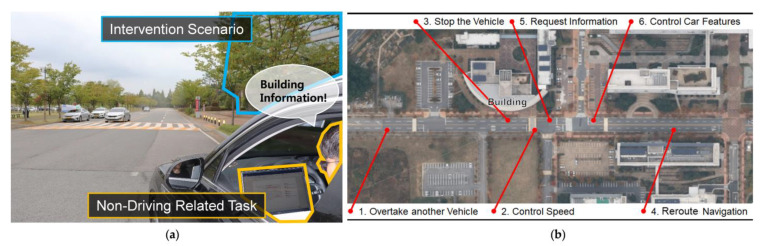
Example snapshot from our video (**a**) showing one intervention scenario (requesting building information) and a driver engaged in an NDRT (working on laptop). (**b**) A map of the routes used to create our survey videos.

We captured our videos by driving on real roads on and near our university campus, as illustrated in Figure 2. Our videos were created using a semiautonomous vehicle (Hyundai Genesis GV80) capable of performing adaptive cruise control with semiautonomous driving mode (Level 2.5), automated emergency braking with pedestrian detection, and lane-departure warning with lane-keeping assist. The vehicle incorporates a machine learning-based smart cruise control that maintains distance by sensors, such as the front camera, and radar from the vehicle in front, while traveling at a speed set by the driver, a core technology for the ADAS. Videos were taken by two GoPro cameras showing the driver’s side view and the vehicle front view inside and outside the vehicle.

In total, 24 videos were processed and presented in the survey (four videos presenting the four NDRT cases for each of six intervention scenarios). The first video (around 2 min) is a combination of seven short clips of the driver’s side view and the car’s front view side-by-side. In each clip, participants were informed about the NDRT (“Relax”) and the corresponding intervention scenario (e.g., overtake the car ahead) and one of seven possible input interactions the driver could use. During each video, the driver’s input interaction was highlighted and the video was briefly paused to allow participants to focus on the type of input interaction used. The videos were designed to allow participants to watch various combinations of input interactions that could fit the FAV environment. The second, third, and fourth videos (15 s each) showed the remaining NDRTs (Table 1). Participants were asked to watch the videos in full screen to increase the feeling that they were driving the FAV and, obviously, to watch all input interactions completely.

In total, we surveyed six intervention scenarios: four maneuver-based interventions and two nonmaneuver-based interventions (Table 2). For each intervention scenario, participants were shown a split-screen video of a driver engaged in an NDRT (e.g., *relaxing*) intervening with the vehicle to perform a request (e.g., overtake the car ahead) using various input interactions (e.g., touch, voice, etc.). The right side of the screen video showed the car front view, which displays the action being performed, while the left side showed the driver requesting the intervention scenario using the assigned input interactions (Figure 1). To minimize the learning effect, we designed a counterbalanced, between-group study design. Each participant answered questionnaires related to only two intervention scenarios and watched their related video scenarios to lessen participant fatigue and frustrations. In total, we created 30 survey copies with a different order of intervention scenarios.

To ensure that participants understood our survey concept and increase data validation, the survey began with an introduction explaining the major differences between FAVs and nonautonomous vehicles with respect to design, possible non-driving-related activities, level of autonomy, and driver interaction possibilities. The driver’s in-vehicle activities were specifically highlighted and shown as examples of what participants could do inside the FAV. To inform the participants of the survey structure, the introduction was followed by a description of the survey steps and goal.

**Figure 3:**
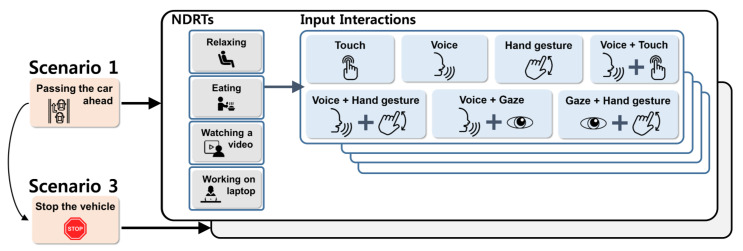
Diagram of our survey implementation; two pairs of the scenario were provided per each participant with a counterbalanced design.

After providing a brief introduction about our survey, participants were asked to answer questions related to two intervention scenarios according to each of the four NDRTs, Figure 3. For each intervention scenario, participants started with the *relaxing* condition, which we considered as a baseline for data collection because it has the lowest physical load and cognitive load. In the *relaxing* condition, we showed participants the two minute video and asked them to rank the seven input interactions from most to least preferred to perform the given intervention scenario.

The ranking question was then followed by asking participants to evaluate each input interaction using a 5-point Likert scale (1—very low, 5—very high) based on 7 parameters: “Familiarity”, “Quick to learn”, “Easy to use”, “Satisfactory”, “Naturalistic”, “Controlling”, “Useful”, and overall participant satisfaction. Previous studies have found that these seven parameters are paramount for general user interface design, particularly for in-vehicle infotainment systems [18,48,63]. Since the evaluation of “Familiarity” and “Quick to learn” parameters are not affected with a change in NDRTs, these parameters where evaluated once in each survey. However, since the evaluation of the other five parameters is subject to change with a change in NDRT, they were evaluated across all NDRT conditions for all input interactions.

- **Familiarity:** I know how to use this input interaction or have used it before.
- **Quick to learn:** I think I can learn this input interaction quickly and easily.
- **Easy to use:** I think it is easy to use this input interaction to request this scenario while performing the current NDRT (e.g., eating).
- **Satisfactory:** I feel satisfied using this input interaction to request this scenario (e.g., to request the overtaking) while performing the current NDRT.
- **Naturalistic:** I think I would feel natural interacting with the car using this input interaction to request the current scenario (e.g., to request the overtaking) while I am involved in the current NDRT.
- **Controlling:** I feel I am optimally controlling vehicle behavior using this input interaction to request this scenario while performing the current NDRT.
- **Useful:** I think this input interaction could match the typical interactions in today’s cars to be used to request the current scenario (e.g., stopping the car!) while performing the current NDRT (e.g., relaxing).

After rating all input interactions in the *relaxing* condition, participants also ranked each input interaction for the given intervention scenario related to other NDRTs (*working on a laptop*, *watching a video*) (Table 1), while following the same sequence: watch a 15 s video, answer the ranking question, rate each input interaction, and describe in words how to perform their most preferred input interaction.

Our task included explicitly verifiable questions to allow us to filter invalid data [30,59]. We removed data that contained irrelevant responses between participant descriptions about the preferred input and their input interaction rankings. This was used to validate the responses if the participant’s description matched their ranking. Although the 1 h completion time was hidden from participants, it allowed us to eliminate responses from participants who were not engaged and did not complete the survey [59]. Following this data filtration of our total gathered responses (*n* = 120) for each scenario, 108 valid responses remained (total 324 responses).

## 3. Results

We explore the results with a hierarchical approach to identify factors in regard to how drivers’ preferences of input interaction are affected by the NDRT and the intervention scenario. Our survey results were filtered with Microsoft Excel then analyzed with Statistical Package for the Social Sciences (SPSS) [64]. Our statistical method includes nonparametric tests such as an independent samples Kruskal–Wallis H test and Friedman’s ANOVA test for the data that were not normally distributed, and parametric tests such as a one-way ANOVA and an independent samples t-test for the data that were normally distributed [65]. Analysis Of Variance (ANOVA) is a group of statistical models used as a method for testing significance of difference between means. Equivalence tests were performed to analyze pairwise comparisons (Bonferroni-corrected or Games–Howell-corrected as post hoc tests) [66].

### 3.1. Identifying Input Interaction Preferences

#### 3.1.1. Input Interaction Preference Order Based on the Ranked Data

Our participants were asked to watch the proposed videos then rank the seven input interactions from 1 (lowest, “this input interaction could be chosen last”) to 7 (highest, “this input interaction could be chosen first”) in each NDRT condition. The average preference of input interaction ranking across all scenarios and NDRTs were assessed to identify overall preference order. The results showed that regardless of NDRT and intervention scenarios, single input interactions ranked higher than multimodal input interactions, where voice input was most preferred.

**Figure 4:**
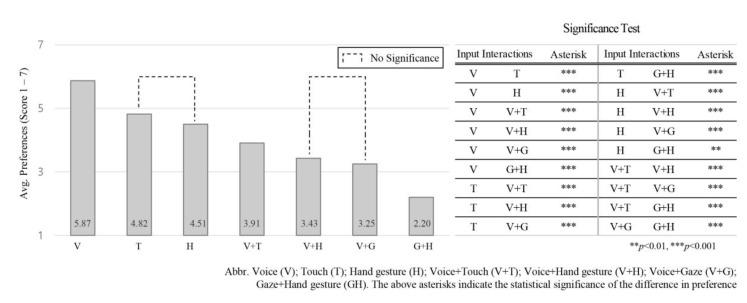
Average preference for seven input interactions according to the “ranking data” across all intervention scenarios and NDRT conditions.

Friedman’s ANOVA test found differences between the tested input interactions to be statistically significant χ2(6) = 542.944, *p* = 0.022, where χ2 value (“chi-square”), (“df”) the degrees of freedom associated with our test statistic, and “*p*” value the significance level. Voice input (*M* = 5.87, *SD* = 0.53) was rated significantly higher than all other input interactions (*p* = 0.000). Participants then preferred touch (*M* = 4.82, *SD* = 0.56), hand gesture (*M* = 4.51, *SD* = 0.58), voice + touch (*M* = 3.91, *SD* = 0.38), voice + hand gesture (*M* = 3.43, *SD* = 0.34), voice + gaze (*M* = 3.25, *SD* = 0.52) and gaze + hand gesture (*M* = 2.20, *SD* = 0.51), in that order, where M indicates the mean value and SD indicates the standard deviation values for the input interaction preference score. The significant differences between these inputs are illustrated in Figure 4, where the high ranked input indicates greater preference.

#### 3.1.2. Input Interaction Preference Order Based on the Five Parameters Rate

After participants ranked the seven input interactions, they were asked to rate each input interaction based on five parameters (easy to use, satisfactory, naturalistic, controlling, and useful) using a 5-point Likert scale (1—very low, 5—very high). To assess each input’s average rating based on the five parameters, we tested the parameters’ overall consistency by finding Cronbach’s alpha to measure internal reliability. Cronbach’s alpha (α) measures how closely related a set of items are as a group, where α greater than 0.7 indicates high internal consistency and reliability. Our results shows that Cronbach’s α = 0.97, which indicates that the average of the parameters has high reliability and are not distinguishable from each other. Consequently, we analyzed the preference order for all inputs based on the five parameters across all NDRTs and intervention scenarios. We found that the preference order based on the five parameters almost same as the preference order based on ranking data. This implies that the selected five parameters are sufficient to reflect participants’ overall ranking of the input interactions and can represent a detailed demonstration of how a user weighs each input internally. Voice input was the most preferable among all inputs because it has the highest rate for all five parameters. Controlling, naturalistic, and easy to use parameters are the main contributor to the preference order of the rest of input interactions.

**Figure 5:**
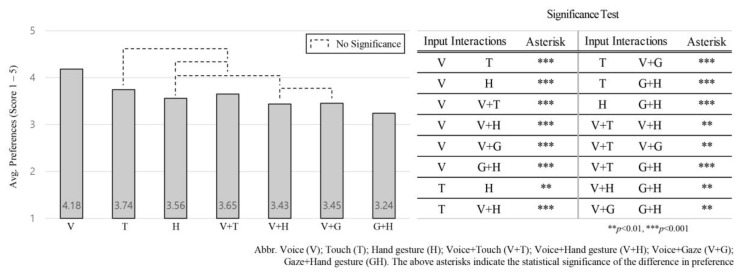
Average preference for the seven input interactions according to “the five parameters” across all intervention scenarios and NDRTs.

The one-way ANOVA test shows significant differences between these input interactions: *F* (6, 742) = 68.23, *p* = 0.000. Voice input was again the most preferred input (*M* = 4.18, *SD* = 0.37) followed by touch (*M* = 3.74, *SD* = 0.34), voice + touch (*M* = 3.65, *SD* = 0.32), hand gesture (*M* = 3.56, *SD* = 0.33), voice + gaze (*M* = 3.45, *SD* = 0.39), voice + hand gesture (*M* = 3.43, *SD* = 0.35), and gaze + hand gesture (*M* = 3.24, *SD* = 0.48), in that order. The preference order of the input interactions in relation to the five parameters changed: Voice + touch with hand gesture inputs and voice + gaze with voice + hand gesture switched preference order. The significant differences between these inputs are illustrated in Figure 5, where the more preferred input interaction scored high for the average of the five parameters.

**Figure 6:**
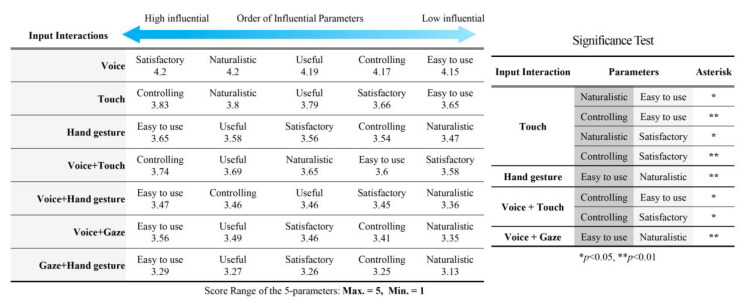
The influence order of five parameters in each input interaction (**left**) and the significance of the parameters in each input interaction (**right**). The deeper blue arrow indicates more influential parameters.

To further investigate the influential parameters for trends and having a detailed description for the parameters that led to this ranking, we assessed the order of the five parameters in each input interaction, Figure 6. The one-way ANOVA test showed significant differences for the rated parameters in the following input interactions: *F* (4, 530) = 5.524, *p* = 0.000 for touch; *F* (4, 530) = 3.496, *p* = 0.008 for hand gesture; *F* (4, 530) = 3.557, *p* = 0.007 for voice + touch; *F* (4, 530) = 3.689, *p* = 0.006 for voice + gaze.

To understand the reason behind the change in the preference order between (hand gesture with voice + touch) and (voice + gaze with voice + hand gesture) inputs, in Figure 5, we analyzed the average rate of the five parameters in these inputs. The ANOVA test was shown that voice + touch was rated significantly high: controlling (*M* = 3. 73, *SD* = 0.36, *p* = 0.000), useful (*M* = 3. 69, *SD* = 0.37, *p* = 0.001), and naturalistic (*M* = 3. 65, *SD* = 0.37, *p* = 0.02), more so than hand gesture, where voice + gaze input was significantly easier to use (*M* = 3. 56, *SD* = 0.34, *p* = 0.04) than voice + hand gesture (*M* = 3. 46, *SD* = 0.35).

### 3.2. Influence of NDRTs on the Input Interactions

In Section 4.1, we discuss the preference order of input interactions regardless of the NDRT; however, in this section, we analyze the influence of NDRT on user preferences and how the overall rank may change. To do that, we calculated the average of the ranked data in each NDRT for all input interactions regardless of the six intervention scenarios.

#### 3.2.1. Preference Order of Input Interactions in Each NDRT

To understand how each NDRT influences input interaction preference order, we assessed the preference order of all input interactions in each NDRT separately regardless of the intervention scenarios. For each NDRT, input interaction ranking was performed using Friedman’s ANOVA test. We found that both voice and gaze + hand gesture inputs kept their preference order position across the change of NDRTs (first and last, respectively), but the preference order of other input interactions changes with the change of NDRT, though none of these changes were significant. For the *watching a video* NDRT, the input interactions preference order matches the case of preference order regardless of NDRTs and the intervention scenarios in Figure 4. Therefore, the NDRT condition affects the preference order as a numerical value but has little practical effect.

**Figure 7:**
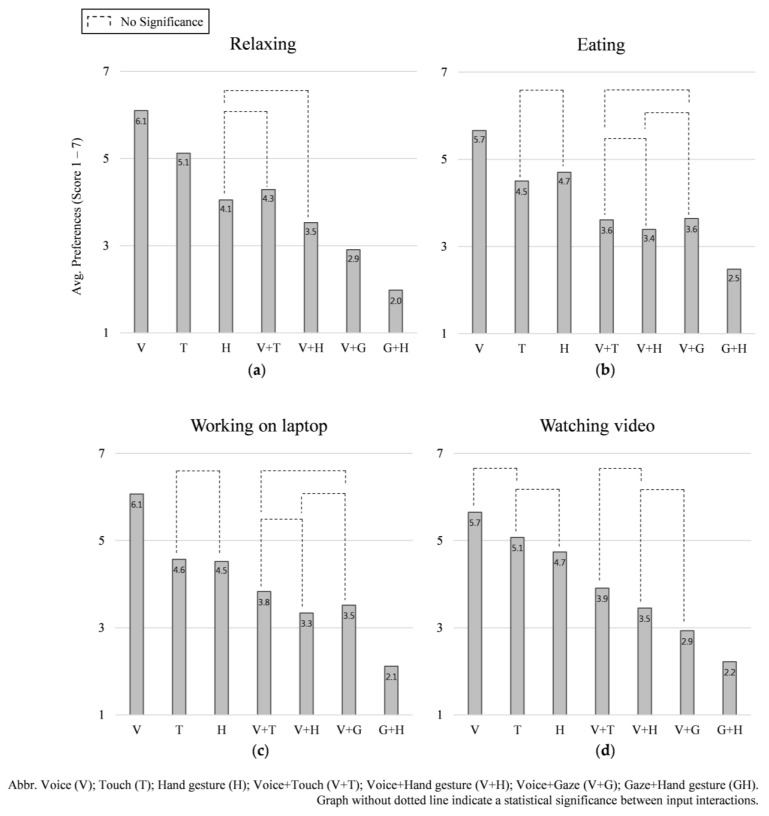
Preference scores for the seven input interactions in the four NDRTs: (**a**) relaxing, (**b**) eating, (**c**) working on laptop, and (**d**) watching video.

In the *relaxing* condition, the ANOVA test shows significant differences overall between input interactions: *χ2*(6) = 504.072, *p* = 0.000. Participants preferred voice input first (*M* = 6.10, *SD* = 0.63) followed by touch input (*M* = 5.12, *SD* = 0.67), voice + touch (*M* = 4.29, *SD* = 0.61), hand gesture (*M* = 4.05, *SD* = 0.72), voice + hand gesture (*M* = 3.53, *SD* = 0.65), voice + gaze (*M* = 2.91, *SD* = 0.64), gaze + hand gesture (*M* = 1.98, *SD* = 0.64), in that order. There were no significant differences in the preference order between hand gesture and voice + hand gesture and between hand gesture and voice + touch input interactions. See Figure 7a.

In the condition, our test shows significant differences overall between input interactions: *χ2*(6) = 387.497, *p* = 0.000. Participants also preferred voice input first (*M* = 5.66, *SD* = 0.81) followed by hand gesture (*M* = 4.70, *SD* = 0.77), touch input (*M* = 4.50, *SD* = 0.86), voice + touch (*M* = 3.61, *SD* = 0.62), voice + gaze (*M* = 3.64, *SD* = 0.89), voice + hand gesture (*M* = 3.39, *SD* = 0.57), gaze + hand gesture (*M* = 2.48, *SD* = 0.78), in that order. There were no significant differences in the preference order between touch and hand gesture, voice + touch and voice + gaze, voice + hand gesture and voice + touch, voice + hand gesture and voice + gaze input interactions. See Figure 7b.

In the *working on laptop* condition, our analysis shows significant differences overall between input interactions: *χ2*(6) = 433.280, *p* = 0.000. Participants preferred voice input first (*M* = 6.07, *SD* = 0.68) followed by touch input (*M* = 4.57, *SD* = 0.79), hand gesture (*M* = 4.52, *SD* = 0.75), voice + touch (*M* = 3.83, *SD* = 0.62), voice + gaze (*M* = 3.52, *SD* = 0.79), voice + hand gesture (*M* = 3.34, *SD* = 0.57), gaze + hand gesture (*M* = 2.12, *SD* = 0.72), in that order. There were no significant differences in the preference order between touch and hand gesture, voice + touch and voice + gaze, voice + hand gesture and voice + touch, voice + hand gesture and voice + gaze input interactions. See Figure 7c.

In the *watching a video* condition, the ANOVA test shows significant differences overall between input interactions: χ2(6) = 476.208, *p* = 0.000. Participants preferred voice input first (*M* = 5.65, SD = 0.73) followed by touch input (*M* = 5.07, *SD* = 0.68), hand gesture (*M* = 4.74, *SD* = 0.79), voice + touch (*M* = 3.91, *SD* = 0.57), voice + gaze (*M* = 3.45, *SD* = 0.52), voice + hand gesture (*M* = 2.93, *SD* = 0.63), gaze + hand gesture (*M* = 2.22, *SD* = 0.71), in that order. There were no significant differences in the preference order between touch and voice, touch and hand gesture, voice + hand gesture and voice + touch, voice + hand gesture and voice + gaze input interactions. See Figure 7d.

#### 3.2.2. Input Interaction Preference Rate across NDRTs

In order to understand how preference of the input interaction is influenced by NDRT, we assessed the average ranking of each input interaction in the four conditions of NDRTs. With the exception of voice + hand gesture, the preference of all input interactions has been highly influenced by the NDRT.

**Figure 8:**
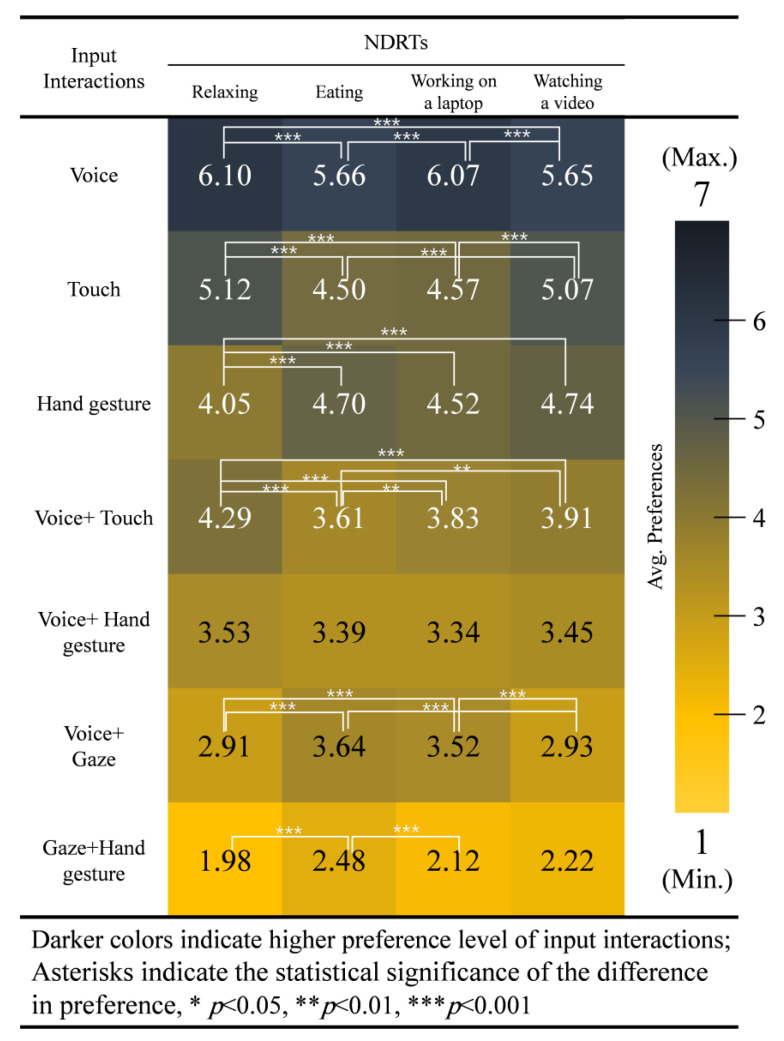
Preference scores for each input interaction associated with the type of NDRT.

A Kruskal–Wallis H test for the average ranked data in each NDRT shows significant differences between NDRTs in these input interactions: voice (*H* (3) = 36.319, *p* = 0.000), touch (*H* (3) = 49.221, *p* = 0.000), hand gesture (*H* (3) = 48.922, *p* = 0.000), voice + touch (*H* (3) = 57.810, *p* = 0.000), voice + gaze (*H* (3) = 73.394, *p* = 0.000), gaze + hand gesture (*H* (3) = 24.614, *p* = 0.000), where there was no significant differences shown in voice + hand gesture (*H* (3) = 6.354, *p* = 0.096), as illustrated in Figure 8.

In detail, participants preferred voice input (*p* = 0.000) in the *relaxing* and *working on a laptop* NDRTs but not in the *eating* and *watching a video* NDRTs. Touch input was significantly preferred (*p* = 0.000) when *relaxing* or *watching a video* compared to when *eating* or *working on a laptop*. Hand gesture was least preferred when *relaxing* compared to *eating* (*p* = 0.000), *working on a laptop* (*p* = 0.000), and *watching a video* (*p* = 0.000). Voice + touch input was preferred when *relaxing* compared to when *eating* (*p* = 0.000), *working on a laptop* (*p* = 0.000), and *watching a video* (*p* = 0.000). Voice + touch input was also preferred when *watching a video* (*p* = 0.001) or when *working on a laptop* (*p* = 0.006) compared to while *eating*. Voice + gaze input was significantly preferred when *eating* (*p* = 0.000) and *when working on a laptop* (*p* = 0.000) compared to when *watching a video* or *relaxing*. Lastly, gaze + hand gesture was significantly preferred when *eating* (*p* = 0.000) compared to when *working on a laptop* or *relaxing*.

### 3.3. Influence of Physical and Cognitive Load

We explored the influence of participants’ physical and cognitive load of performing NDRTs on preference of the input interaction regardless of intervention scenario. We grouped the NDRTs according to load level (high or low) for testing the physical load and another two groups in testing the cognitive loads. In the case of physical load, for example, *eating* and *working on a laptop* NDRTs were combined for high physical load, and *relaxing* and *watching a video* were combined for low physical load. We similarly combined high cognitive load (*working on a laptop* with *watching a video*) NDRTs and for the low cognitive load (*relaxing* with *eating*) NDRTs, Table 1.

#### 3.3.1. High Physical Load vs. Low Physical Load

**Figure 9:**
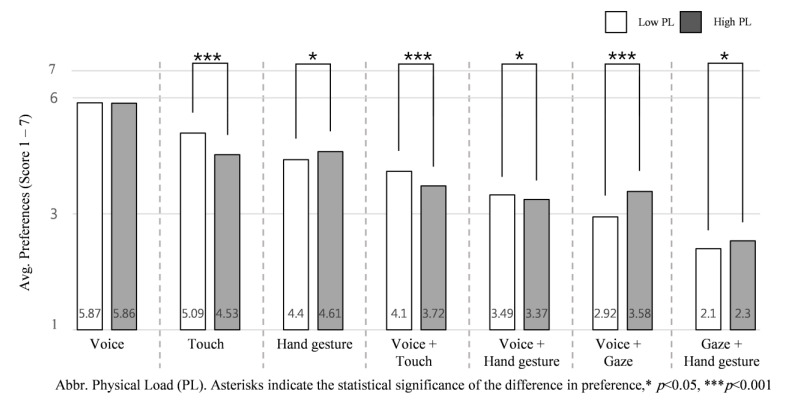
Average preference for the seven input interactions according to physical load of NDRTs: low physical load vs. high physical load.

Assessing the average ranking of each input interaction according to low and high physical load NDRTs across all the intervention scenarios helps us understand how engagement in high physical load NDRTs influences participant preference of input interaction. Participants’ responses revealed that changes in physical load of NDRTs influences the preference order of input interactions, with the exception of voice input, as shown in Figure 9.

The Kruskal–Wallis test shows participants significantly prefer to use some input interactions while engaged in low physical load NDRTs: touch input (*M* = 5.09, *SD* =.55), *H* (1) = 30.962, *p* = 0.000, voice + touch (*M* = 4.10, *SD* = 0.46) *H* (1) = 27.979, *p* = 0.000, and voice + hand gesture (*M* = 3.49, *SD* = 0.43) *H* (1) = 5.640, *p* = 0.018. On the other hand, participants significantly preferred the following input interactions when engaged in high physical load NDRTs: hand gesture (*M* = 4.61, *SD* = 0.64), *H* (1) = 5.845, *p* = 0.016, voice + gaze (*M* = 3.58, *SD* = 0.73), *H* (1) = 46.912, *p* = 0.000 and gaze + hand gesture (*M* = 2.30, *SD* = 0.65) *H* (1) = 4.277, *p* = 0.039.

#### 3.3.2. High Cognitive Load vs. Low Cognitive Load

We assessed the average ranking of each input interaction according to low and high cognitive load NDRTs across all intervention scenarios in order to understand how engagement in high cognitive load NDRTs influences participant preference of input interaction. We found that, unlike physical load, the cognitive load of NDRTs slightly impacts preference of input interactions, specifically in regard to hand gesture.

**Figure 10:**
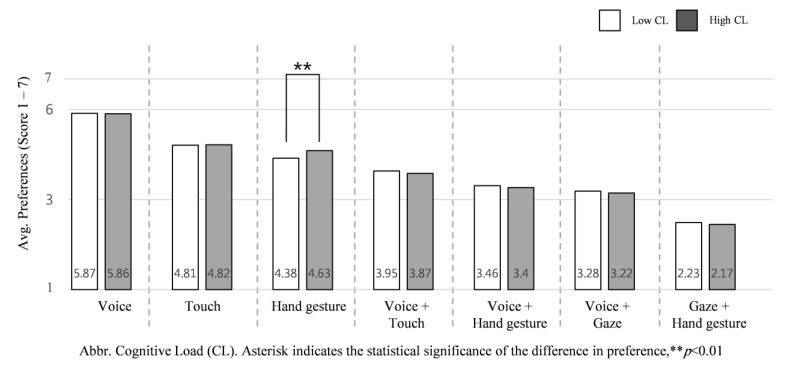
Average preference for the seven input interactions according to cognitive load of NDRTs: low cognitive load vs. high cognitive load.

When cognitive load is high, the Kruskal–Wallis test shows hand gesture (*M* = 4.63, *SD* = 0.68), *H* (1) = 10.052, *p* = 0.002 was the only input significantly preferred. Compared to their physical load, the cognitive load of NDRTs influences input interaction preferences relatively little, as shown in Figure 10.

### 3.4. Influence of Intervention Scenarios (MBI vs. NMBI)

**Figure 11:**
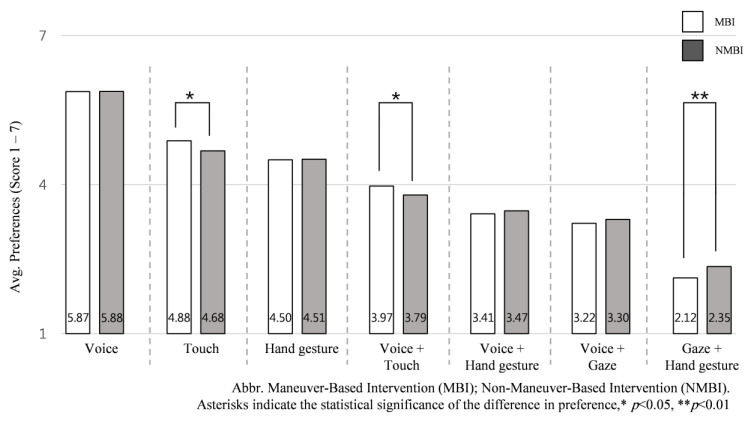
Average preference for the seven input interactions according to the intervention scenarios: maneuver-based intervention (MBI) vs. nonmaneuver-based intervention (NMBI).

To explore further the influential factors on the preference of input interactions and understand how they differ between intervention scenarios, we assessed the average ranking of each input interaction in MBI and NMBI scenarios across all NDRTs. To do this, the six intervention scenarios were classified within two groups, a Maneuver-Based Intervention (MBI; scenario 1, 2, 3, 4) and Nonmaneuver-Based Intervention (NMBI; scenario 5, 6). Our finding shows that regardless of NDRTs, MBI and NMBI scenarios influence input interaction preferences. Touch-based inputs were highly preferred in MBI scenarios, and gaze + hand gesture was preferred in NMBI, see Figure 11.

The independent-samples Kruskal–Wallis H test found that two input interactions were significantly preferred in MBI scenarios compared to NMBI scenarios, touch input (*M* = 4.95, *SD* = 0.56), *H* (1) = 4.548, *p* = 0.033 and voice + touch input (*M* = 3.99, *SD* = 0.47), *H* (1) =6.445, *p* = 0.011, where participants significantly preferred gaze + hand gesture (*M* = 2.40, *SD* = 0.85), *H* (1) = 7.328, *p* = 0.007 in the NMBI scenario rather than MBI scenario.

## 4. Discussion

Our results show that preference order of the proposed input interactions was influenced by the type of NDRT and the intervention scenarios. In this section, we discuss four parts based on our research questions and offer a decision-making model diagram to help understand how drivers select an input interaction to intervene in FAVs.

### 4.1. Evaluate the Proposed Natural Input Interactions

In our survey, participants were asked to rank all input interactions from 1 to 7 for each NDRT condition. The overall average of the proposed input interactions was assessed regardless of NDRT and intervention scenarios to understand the preference order of the input interactions across all NDRTs and intervention scenarios. The rank of the proposed input interactions is voice, touch, hand gesture, voice + touch, voice + hand gesture, voice + gaze, and gaze + hand gesture. Voice input interaction ranked first, which makes it the most intuitive and naturalistic input interaction regardless of NDRT and intervention scenario category. This result confirms findings in previous studies where voice input ranked first for FAV use compared to touch input [32] and compared to hand gesture input [16]. In contrast, gaze + hand gesture ranks last. This does not mean it is the worst input interaction, however, as participants may prefer to use it in specific circumstances, as we discuss later.

Evaluating the input interaction according to user rank gives an absolute preferable value. Therefore, after ranking, participants were asked to rate each input interaction based on five parameters (easy to use, satisfactory, naturalistic, controlling, and useful). However, to make sure that our parameters covered all participants’ perceptions in relation to scoring each input interaction, we assessed the order of the input interactions according to the average of five parameters across all NDRTs and intervention scenarios, as shown in Figure 5. The overall preference order for input interactions based on the given five parameters: voice; touch; voice + touch; hand gesture; voice + gaze; voice + hand gesture; and gaze + hand gesture, has two differences compared to the order based on the absolute ranking scale shown in Figure 4. In the absolute rankings, participants ranked hand gestures and voice + touch as the third and fourth most-preferred input interactions, respectively. When the five parameters are taken into account, participants switched that order: voice + touch ranked third and hand gestures ranked fourth as most-preferred input interaction. Similarly, in the absolute rankings, voice + hand and voice + gaze ranked fifth and sixth overall, respectively, yet when the five parameters are taken into account, their respective rankings traded places.

Even though the reversal in the order preferences of theses inputs (hand gesture with voice + touch and between voice + hand gesture with voice + gaze) occurred when evaluated by using the five parameters, in general there are no significant differences between them. This implies that the parameters used to evaluate our input interactions were efficient and covered participants’ rating aspects.

To better understand why the preference order of theses inputs changed and what parameters contributed to that change, we assessed the influence of each parameter between these inputs, as shown in Figure 6. We found that voice + touch ranked higher than hand gesture because it was shown as significantly higher and more naturalistic, controlling, and useful, whereas the easy to use and satisfactory parameters were relatively even between the two input interactions. Similarly, voice + gaze ranked higher than voice + hand gesture because it was shown as significantly higher and more easy to use than voice + hand gesture input.

To conclude, voice input interaction was the most preferred input in all cases because it rates highest in all five parameters and shows no significant difference between them. The preference order for the other input interactions were mainly influenced by the controlling, naturalistic, and easy to use parameters. This implies that controllability, naturalness, and ease of use are the main factors to consider while designing or suggesting an input interaction.

Since preference order based on the five parameters was almost the same as the preference order based on ranking data, the following analysis is based on the ranking data.

### 4.2. Effect of NDRT on the Selection of Input Interaction Regardless of Intervention Scenarios

In order to understand how input interaction preference is influenced by NDRT regardless of intervention scenario, participants were asked to rank the seven input interactions in the four conditions of NDRTs according to their preferences.

For each NDRT, we assessed the preference order of all input interactions. Even though the preference order changed according to NDRT, single input interactions were more preferred than multimodal input interactions in almost all cases; the lone exception is that participants preferred voice + touch input to hand gesture input during the *relaxing* condition, Figure 7. Moreover, the preference order of input interactions for each NDRT largely mirrored the overall preference order of input interactions, independent of NDRT and intervention scenario, see Figure 4. To further investigate how preference of input interaction is influenced by NDRT, we assessed each input interaction according to the four conditions of NDRTs, Figure 8.

- **Voice input:** Participants significantly preferred voice input when *relaxing* or *working on a laptop* than when *eating* or *watching a video*. Indeed, voice input was known to minimize distraction [34] and require minimum visual attention [21]. Conversely, voice input disrupts driver attention when another sound source is active in the FAV, like when *watching a video*, and is inconvenient when the driver’s mouth is busy. This confirms findings in [16,34].
- **Touch input:** Participants significantly preferred touch input while *relaxing* or *watching a video* than while *eating* or *working on laptop*. Unsurprisingly, touch input is preferred more when the NDRT does not require the use of hands. In other words, since the use of touch input increases physical load, drivers prefer not to use it while they are engaged in high physical load NDRTs.
- **Hand gesture:** Participants preferred hand gesture while *watching a video*, *eating*, and *working on a laptop* significantly higher than when *relaxing*. Indeed, when *relaxing*, drivers would not want to initiate physical activity like hand movement and risk their relaxation. However, drivers are physically active when *eating* or *working on a laptop*, which explains why hand gesture would be easier to use as an input interaction for those NDRTs.
  While *watching a video*, hand gestures seem like the least obtrusive option; i.e., drivers prefer not to avert their gaze from the screen, give a voice command, or lean forward to perform touch input. Therefore, for this NDRT, hand gesture could be an ideal candidate.
- **Voice + Touch:** Participants preferred voice + touch when *relaxing* significantly higher than in all other NDRTs. However, using voice + touch input while *eating* ranked significantly lower than using it when *working on laptop* or *watching a video*. Participants still do not prefer to use voice inputs while *eating*, and adding touch to voice would increase physical load for the driver. Therefore, voice + touch input interaction is preferable while *relaxing* mainly, though not as preferable as voice alone. Although an input interaction that includes voice would seem counterintuitive to *watching a video*, a single word could be used to trigger an interaction, which the driver could complete using touch input. Therefore, it also scores relatively high while engaging in *watching a video*.
  Tscharn et al. [17] found that voice + touch input causes a physical and cognitive load and redirects attention from the driver’s current view to the touch panel to interact. Thus this input was preferred only while *relaxing* which has no high physical or cognitive load and nothing to be distracted from. On other hand, it was not preferable while *eating*, *working on laptop*, or *watching a video*, NDRTs that require driver attention. Furthermore, each of these NDRTs cause high physical or cognitive load, which make an input that increases them not preferable.
- **Voice + Hand gesture:** Participants reported no significant differences in using voice + hand gesture between the four NDRTs. Thus, using hand gesture input could be considered as a neutral input between the four NDRTs where no significant difference in usage could be noticed. This finding confirms [17], where voice + hand gesture was most preferred and shown as more naturalistic than voice + touch input for intervening in the FAV.
- **Voice + Gaze:** Participants preferred to use voice + gaze while *eating* or *working on a laptop* significantly higher that when *relaxing* or *watching a video*. Although voice input alone was not preferred to use while *eating*, combining gaze with voice interaction compensates for the drawbacks of using voice input alone; e.g., drivers could use gaze to select an intended object while chewing, then use voice to confirm the selection via a trigger word between bites. This decreases the physical load voice input causes in talking which make its usage while *eating* or *working on* a *laptop* preferable. On the other hand, it may not work well while *watching a video*, where any voice commands and diversion of gaze would interrupt the experience or while *relaxing* where drivers would not want to initiate physical activity like speaking or moving their eyes and risk losing their *relaxing* state.
- **Gaze + Hand gesture**: Participants preferred gaze + hand gesture while *eating* significantly higher than while *relaxing* or *working on laptop*. Again, gaze input is not preferred while *relaxing*, yet seems helpful when the participant is engaged in a high physical load task like *eating* because drivers are physically active. This matched with the case of using hand gesture alone. It was also less preferable while *relaxing*.

Each input interaction is best suited for one situation. Voice input, for example, is preferred when there is no sound in the car, whereas touch input and hand gesture input are not preferred in high physical load and low physical load activities, respectively. This implies that NDRT highly influences preference of input interaction.

#### Physical and Cognitive Load Variation Effect

We explored the influence of participants’ physical and cognitive load of performing NDRTs on preference of the input interaction regardless of intervention scenario.

To identify the influence of physical load of NDRT on the proposed input interactions, we assessed average preference by using each input interaction in high physical load and low physical load of NDRT conditions, as shown in Figure 9. Participants highly preferred to use hand gesture, voice + gaze, and gaze + hand gesture inputs while engaged in a high physical load compared to low physical load tasks, where touch, voice + touch and voice + hand gesture input interactions while engaged in a low physical load compared to high physical load tasks. Referring to the finding discussed in Section 4.2, since gaze input interaction causes driver distraction [21], participants did not prefer to use any gaze-based input interactions while they are engaged in *relaxing* or *watching a video* (low physical load NDRTs); this causes its presence in the high physical load. In addition, hand gesture input was preferred when the participant’s body was physically active, which causes its preference while engaged in high physical load NDRTs.

To identify the influence of cognitive load of NDRT on the proposed input interactions, we assessed average preference of using each input interaction in high cognitive load and low cognitive load of NDRT conditions, as shown in Figure 10. Since hand gesture was shown as a lower cognitive load input in a previous study [21] and was significantly preferred when *watching a video* and *working on a laptop* (high physical load NDRTs), it is significantly preferred by drivers involved in a high cognitive load task.

In summary, in high physical load and high cognitive load NDRTs, hand gesture could be a strong candidate. In addition, voice input was the only input interaction that showed no significant differences with varying low or high physical and cognitive load, whereas the preference of the other input interactions changed based on the physical load levels only.

### 4.3. Effect of Intervention Scenario Category

To understand the influence of the intervention scenarios on the preference rate of input interactions, we assessed the average preference of using each input interaction in maneuver and nonmaneuver-based intervention scenarios. Since MBI scenarios are highly related to vehicle operation, including lateral and longitudinal controls, and align with driver’s safety and increase cognitive load (compared to NMBI scenarios, which are related to vehicle infotainment system and do not increase cognitive load), MBI can be described as a critical intervention and NMBI scenarios as a noncritical intervention. Previous work [16] suggested that input interaction for the MBI should offer high controllable input, low input errors, and short input times. Therefore, participants prefer trusted input interactions to perform MBI, such as touch and voice + touch. Generally, we found touch-based input interactions are more trusted by participants, who rated both inputs as highly controllable and familiar, as shown in Figure 6. Consequently, they prefer to use touch-based inputs for more critical interventions. On the other hand, participants prefer gaze + hand gesture input, which are less controlling, for noncritical interventions such as requesting information or operating car features (e.g., running music, or controlling air conditioner) than for critical interventions in FAVs.

Most input interactions could be used for both MBI and NMBI scenarios. Voice input interaction scored the highest in the preference rate for both MBI and NMBI scenarios, while touch inputs were more preferred for MBI scenarios and gaze-hand gesture was more preferred in NMBI scenarios. Overall, after checking the proposed factors that affect the selection of input interaction, we can find that:

1. Different types of NDRTs and accompanying variation in the levels of physical and cognitive load primarily influence the selection of input interactions.
2. Different categories for intervention scenarios (maneuver and nonmaneuver-based interventions) are secondary factors that influence the selection of input interactions.

### 4.4. Driver’s Decision-Making Model for Naturally Intervening FAV

Our aforementioned findings can be synthesized through hypothesizing a decision-making model diagram (inspired by [67,68]) by which people subconsciously determine the most naturalistic ways to intervene in the vehicle system in an instant, tailored to the level of experienced physical and cognitive workload, then rationally compromised to fit the intervention demand and driver preference in FAVs [67,69].

**Figure 12:**
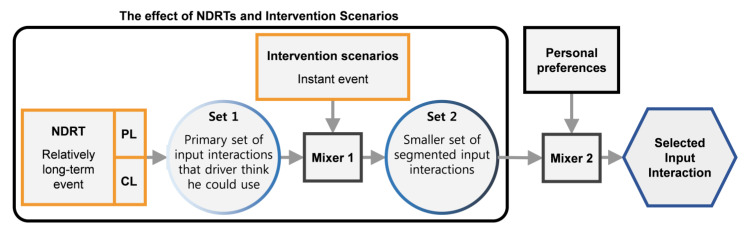
Driver’s decision-making model to intervene in the FAV.

NDRTs could be a relatively long-term event if the driver keeps performing it for a prolonged period while driving. As shown in Figure 12, each NDRT could be classified to a smaller component (physical and cognitive load), wherein the level of each affects significantly the set of input interactions the driver could utilize (Set 1). In FAVs, the driver’s desires trigger the intervention stage where the driver seeks to perform an MBI or NMBI scenario. The category of the intervention scenario has a job similar to the saturation function, which defines whether to select a highly familiar and trusted input interaction to perform the MBI or a less familiar and trusted input interaction to perform the NMBI from Set 1. The output of this stage is a segmented set of input interactions (Set 2) which fit most the intervention category and the physical and cognitive load levels. One input interaction could be picked from Set 2 depending on personal choice. Considering that, the driver has many input interactions to accommodate the designated level of NDRT and the existing intervention scenario. Therefore, the driver’s personal choice selection should inform the selection of available input interactions from Set 2 [24].

#### FAV Suggesting Input Interactions

The model above could be inversely used to develop a way for FAVs to regulate the selection of input interactions. As the driver’s physical and cognitive load could be observed by the FAV (e.g., using image processing and AI techniques), the vehicle will then predict the Set 1 input interactions that are suitable for the driver’s condition. The vehicle could not confirm the exact intervention scenario, but it could prepare two smaller segmented sets from Set 1, one segmented set for MBI scenarios and another for NMBI scenarios. Learning driver behavior and preferences over time will assist the vehicle in the second elimination stage (Mixer 2). The point from this discussion is to clarify that advancements in FAV technology could enable the vehicle to suggest the best input interactions instantaneously and dynamically during the trip (e.g., display most suited input interactions over the dashboard). These suggestions can guide the driver to intervene in the FAV system quickly and comfortably without need to change position or disengage from the NDRT.

### 4.5. Limitations

Our study covered both maneuver- and nonmaneuver-based intervention scenarios, single and multimodel input interactions, and physical and cognitive load NDRTs. Intervention scenarios, input interactions, and NDRTs were selected based on previous studies [4,5,12,17] that showed that these functions and features are the most frequently used and common in actual driving situations. However, other scenarios, inputs, and NDRTs would be worth studying, such as adjusting driver’s seat or asking the FAV to exit the highway, using a button input, or using a smartphone NDRT.

Although researchers have implemented online survey-based videos for AV studies [30,31], which our survey followed, there are still limits in replicating an in-vehicle experience. In-vehicle studies with a real AV are needed to investigate how well participants’ online responses reflect their experience in real-life situations and how other real-world factors may also influence driver acceptance and preferences for input interactions.

This study shows the results across participant age groups; however, discriminating between age groups may show more interesting results.

Roughly 80% of our participants were from United States; the remaining ~20% of participants were from India, UK, Egypt, and Brazil. Distributing our survey across a larger geographical area may give us clearer information about how different cultural backgrounds may affect driving intervention behavior in FAVs.

## 5. Conclusions and Future Work

We conducted an online video-based survey, where we focus on the relatively new environment for the FAV to determine the most possible natural input interactions that match the driver’s in-vehicle activities and the interaction situation. The preference order of seven input interactions was evaluated while considering different physical and cognitive loads of NDRTs through various possible intervention scenarios in order to investigate the factors that influence driver input interaction preferences. Regardless of NDRT and intervention scenarios, single input interactions were generally more preferred than multimodal inputs, yet participants preferred multimodal inputs when they are in a resting condition, reclining, and looking forward. Moreover, voice input interaction was the most preferred among all input interactions. The preference order for the other input interactions were mainly influenced by the controlling, naturalistic, and easy to use parameters.

Driver engagement in an NDRT was the primary factor influencing interaction input preferences; secondarily, preference of input interaction was influenced by the intervention scenario. The effect of cognitive load of NDRTs influences choice of input interaction less than the effect of physical load of NDRTs. Hand gesture could be a strong candidate for high physical load and high cognitive load NDRTs. In addition, voice input was the only input interaction that showed no significant differences with varying low or high physical and cognitive load.

Understanding the factors that influence driver preference of natural input interaction can help researchers to develop better FAV user interfaces and enhance user experience by considering drivers’ needs and design requirements. Therefore, we presented a driver’s decision-making model to describe the driver’s selection process that helps to promote better design strategy for input interaction in a FAV and suggests more pathways in which a FAV could regulate a driver’s interactions.

We aim to continue this study with more scenarios and more physical and cognitive load NDRTs to understand common and special causes across contexts that influence participant preferences of input interactions.

In future work, as a confirmation stage for this study, we will create an experiment that simulates the FAV environment using a driving simulator or a real autonomous vehicle where participants will be involved physically in NDRTs with the proposed intervention scenarios and the same set of natural input interactions. Afterwards, we could implement the concept where a FAV could regulate the driver’s interactions by constantly updating the proper interaction channels for the driver according to the suggested driver–decision-making diagram. Discriminating between ages in future work, however, may show otherwise; i.e., the cognitive load of NDRTs may show greater influence for older participants than younger participants.

## Figures and Tables

**Table 1:** Description of Non-driving Related Tasks (NDRTs) and condition distribution for physical and cognitive load.

NDRTs (1–4)	Description	Physical Load	Cognitive Load
**Relaxing**	The driver was in a resting condition, reclining and looking forward.	Low	Low
**Eating**	The driver was holding a food container in one hand and an eating utensil in the other.	High	Low
**Working on a laptop**	Using both hands, the driver was typing on a laptop located on his lap.	High	High
**Watching video**	The driver deeply engaged in watching a video playing from the in-vehicle touch screen.	Low	High

**Table 2:** Description of the driver intervention scenarios.

Category	Scenario	Description
Maneuver-based Intervention Scenarios	1- Overtake another Vehicle	Ask the vehicle to pass the car ahead for some reason (e.g., blocks the front view).
	2- Control Speed	Ask the vehicle to slow down then maintain the lower speed.
	3- Stop the Vehicle	Ask the vehicle to stop at a stated location.
	4- Reroute Navigation	Ask the vehicle to reroute the navigation.
Nonmaneuver-based Intervention Scenarios	5- Request Information	Ask the vehicle to provide information about a point of interest.
	6- Control Car Features	Control the vehicle infotainment, e.g., play a YouTube video.

## Data Availability

The data presented in this study are available on request from the corresponding author.
